# Complete Chloroplast Genome Sequence and Phylogenetic Analysis of the Tibetan Medicinal Plant *Soroseris hookeriana*

**DOI:** 10.3390/genes17010024

**Published:** 2025-12-27

**Authors:** Tian Tian, Xiuying Lin, Yiming Wang, Jiuli Wang

**Affiliations:** 1 College of Ecological Environment and Resources, Qinghai Minzu University, Xining 810007, China; ygsn2705@126.com (T.T.); lxy0105200607@outlook.com (X.L.); 2018038@qhmu.edu.cn (Y.W.); 2 Qinghai Provincial Key Laboratory of High Value Utilization of Characteristic Economic Plants, Qinghai Minzu University, Xining 810007, China; 3 Key Laboratory of Resource Chemistry and Eco-Environmental Protection in Qinghai-Tibet Plateau, Qinghai Minzu University, Xining 810007, China; 4 State Key Laboratory of Tibetan Medicine Research and Development, Qinghai University, Xining 810016, China

**Keywords:** *Soroseris hookeriana*, chloroplast genome, phylogeny, evolution

## Abstract

**Background/Objectives**: *Soroseris hookeriana*, a Tibetan medicinal plant endemic to the high-altitude Qinghai–Tibet Plateau, possesses significant pharmacological value but lacks fundamental genomic characterization. This study aims to generate and comparatively analyse its complete chloroplast genome. **Methods**: Total DNA was sequenced, assembled with GetOrganelle, annotated with CPGAVAS2, and compared with eight Asteraceae species; phylogenetic placement was inferred with IQ-TREE from 21 complete plastomes. **Results**: The circular chloroplast genome is 152,514 bp with a typical quadripartite structure (LSC 84,168 bp, SSC 18,528 bp, two IRs 24,909 bp each). It contains 132 unique genes (87 protein-coding, 37 tRNA, 8 rRNA; 18 duplicated in IRs yield 150 total copies). Twenty-three genes harbour introns; *clpP* and *ycf3* have two. Overall GC content is 37.73%, elevated in IRs (43.12%). Codon usage shows strong A/U bias at the third position; 172 SSRs and 39 long repeats are detected. IR-SC boundaries exhibit the greatest inter-specific variation, notably in *ycf1* and *ndhF*. **Conclusions**: The complete plastome robustly supports *S. hookeriana* and *Stebbinsia umbrella* as sister species (100% bootstrap) and provides essential genomic resources for species identification, population genetics, and studies of high-altitude adaptation.

## 1. Introduction

The genus *Soroseris* Stebbins (tribe Cichorieae, Asteraceae) is endemic to the Himalayan Mountains, where species endure extreme environmental conditions including intense solar radiation, hypobaric atmosphere, and pronounced diurnal temperature fluctuations. The genus comprises eight species, all native to China. As a Sino-Himalayan Mountain endemic genus, *Soroseris* occupies narrow alpine niches that may be vulnerable to climate change, making it a candidate for future ecological monitoring studies [1]. Current research on *Soroseris* has primarily focused on pharmacological activities and systematic studies [2]. In 2000, Meng et al. first isolated two novel compounds from *S. hookeriana* subsp. *erysimoides*: a sesquiterpene lactone (3β,8β-dihydroxy-11αH-guaia-4(15),10(14)-diene-12,6α-olide) and a monoterpenoid ((1R,4R,5R)-5-benzoyloxybornan-2-one). Both compounds exhibited significant inhibitory effects against various bacteria and fungi, with the monoterpenoid showing particularly strong activity against Bacillus subtilis. These findings provide promising leads for natural antimicrobial drug development [3]. While *Soroseris* species are known to be diploid or tetraploid, fundamental genomic characteristics of the genus remain unreported.

Chloroplasts are among the most essential organelles in plants, possessing their own genetic system that is vital for photosynthesis and carbon fixation [4]. In land plants, the cp genome is highly conserved in structure and composition, with a characteristic quadripartite architecture: a large single-copy (LSC) region, a small single-copy (SSC) region, and two inverted repeat (IR) regions [5]. The genome encodes approximately 110–130 genes, functionally categorized into three groups: photosynthesis-related genes, genes related to chloroplast gene expression, and open reading frames (ORFs) along with other protein-coding genes [6]. Characterized by low molecular weight, moderate sequence variation, and a conserved yet polymorphic nature, the cp genome offers a simplified system for investigating gene evolutionary patterns and holds significant value for species identification and phylogenetic studies of related taxa [7,8]. Chloroplast genomes have been successfully used to resolve maternal ancestry in domesticated chrysanthemums [9] and to reveal evolutionary history in crop plants like pea [10], demonstrating their power for phylogenetic and population genetic studies. Given the widespread genome rearrangements and intron variation across multiple angiosperm lineages [11,12], the draft assembly of the *S. hookeriana* cp genome will help elucidate the intrinsic links between its chloroplast function, environmental adaptability, and phytochemical properties.

Within the genus *Soroseris*, *S. hookeriana* is the most widely distributed species and a valued Tibetan medicinal herb [13]. The whole plant is traditionally used to treat food poisoning and associated symptoms including fever, headache, scalp lesions, and serous fluid accumulation in the thoracic cavity and limb joints [14]. Despite its significant pharmacological importance, the chloroplast genomic architecture of this species remains uncharacterized. Here, we report the first complete chloroplast genome sequence of *S. hookeriana*, detailing its structural organization, gene content, and IR boundary dynamics. Comparative genomic analyses with selected Asteraceae species illuminate its plastome architecture, while phylogenetic reconstruction clarifies its evolutionary placement within the family. These genomic resources establish a critical foundation for species authentication, population genetics, and sustainable development of *S. hookeriana* as a medicinal resource.

## 2. Materials and Methods

### 2.1. Sample Collection, DNA Extraction, and Sequencing

**Figure 1:**
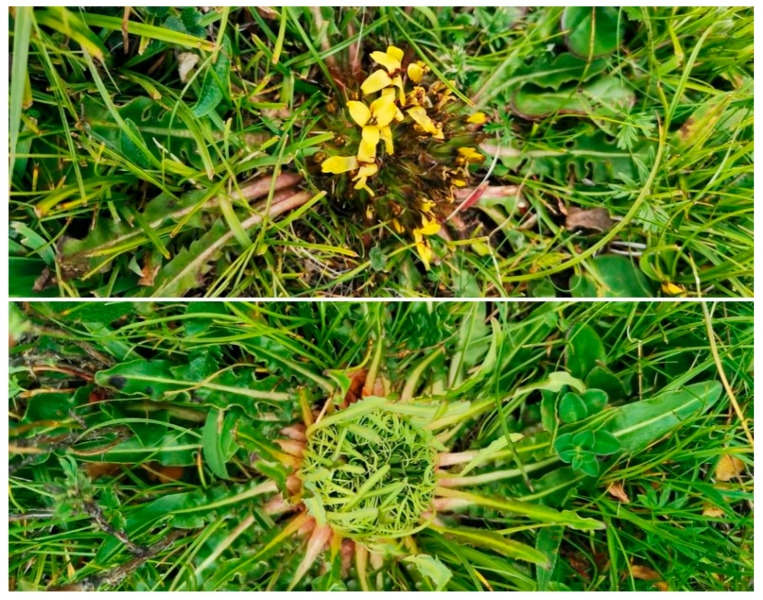
Wild growth of *S. hookeriana*.

Plant material was collected on 27 July 2021, from Lajia Town, Maqin County, Guoluo Tibetan Autonomous Prefecture, Qinghai Province, China (Figure 1; 34°31′ N, 100°57′ E, 4060 m altitude). The specimen was identified as *S. hookeriana* by Dr. Jiuli Wang and is deposited at the Northwest Institute of Plateau Biology, Chinese Academy of Sciences. Total genomic DNA was extracted from fresh leaves using a modified CTAB (cetyltrimethylammonium bromide) method [15]. DNA quality was assessed by 1% agarose gel electrophoresis, and purity and concentration were measured using a NanoDrop 2000c spectrophotometer (Thermo Fisher Scientific, Waltham, MA, USA). Qualified DNA samples were sent to Nanjing Genepioneer Biotechnologies Co., Ltd. (Nanjing, China) for library construction according to the manufacturer’s standard protocols (Detailed protocol parameters are available upon request). After quality control, paired-end (PE) sequencing was performed on an Illumina NovaSeq 6000 platform (Illumina, San Diego, CA, USA) with a read length of 150 bp.

### 2.2. Genome Assembly and Annotation

Raw reads were filtered using fastp (version 0.20.0, Open Gene Foundation, Shenzhen China) to obtain high-quality sequences [16]. Utilizing the default settings of GetOrganelle (v1.7.5) [17] along with the specified parameter “-R 15 -k 21,45,65,85,105 -F embplant_pt”, the high-quality sequences were processed to generate a circular chloroplast genome of *S. hookeriana*. For the annotation of this chloroplast genome, the CPGAVAS2 (version 2.0) tool was employed [18]. Any inaccuracies detected in the annotations of individual genes were subsequently corrected manually through the application of CPGView (http://www.1kmpg.cn/pmgview) [19]. The final annotated genome sequence was then deposited in GenBank under the accession number OM935750.1. Additionally, a chloroplast genome map was constructed using Chloroplot (https://irscope.shinyapps.io/Chloroplot/, accessed 20 December 2025; version not specified) [20].

### 2.3. Analysis of Codon Usage Bias

Codons are fundamental carriers of genetic information for transcription and translation, and their usage bias significantly influences protein translation efficiency and exogenous gene expression [21]. This molecular adaptation mechanism reflects evolutionary responses to environmental changes. The study of codon usage bias in cp genomes facilitates the development of robust chloroplast transgenic systems and elucidates plant genetic evolution and phylogeny [22]. Due to codon degeneracy, multiple synonymous codons can encode the same amino acid, and their non-random usage pattern is termed codon usage bias (CUB). To characterize this bias, relative synonymous codon usage (RSCU) values were calculated for the *S. hookeriana* cp genome.

### 2.4. Repeat Sequence Analysis

Repeat sequences contribute to cp genome rearrangement and enhance population genetic diversity [23]. The Microsatellite identification tool (MISA) (IPK, Gatersleben, Germany) was used to identify simple sequence repeats (SSRs) in the *S. hookeriana* cp genome. The minimum threshold parameters were set as follows: mononucleotide repeats ≥ 8, dinucleotide repeats ≥ 5, and tri-, tetra-, penta-, and hexanucleotide repeats ≥ 3.

### 2.5. Comparative Genomic Analysis

Eight Asteraceae species were retrieved from GenBank for comparative analysis based on three criteria: (1) phylogenetic proximity to *S. hookeriana* within subfamily Cichorioideae and tribe Cichorieae, spanning four subtribes (Crepidinae, Hyoseridinae, Lactucinae) for intratribal comparison; (2) inclusion of *S. umbrella*, the only congeneric species with a published cp genome; and (3) *Aster farreri* (subfamily Asteroideae) as an outgroup. The selected genomes were: *S. umbrella* (MN822134.1), *Sonchus brachyotus* (NC_058614.1), *S. oleraceus* (NC_048452.1), *Paraixeris denticulata* (MK622902.1), *A. farreri* (OQ603808.1), *Ixeris repens* (NC_057108.1), *Faberia sinensis* (NC_066728.1), and *Lactuca sativa* (NC_007578.1). Comparative analysis was performed using the Shuffle-LAGAN mode in mVISTA (https://genome.lbl.gov/vista/mvista/submit.shtml, accessed 20 December 2025; version not specified) [24,25].

In the process of genome evolution, inverted repeat (IR) boundaries are prone to expansion or contraction, which may cause the transfer of specific genes between IR regions and single-copy regions. To determine if there are changes in the size of the boundaries of the four genomic regions. IRSCOPE software [26] (https://irscope.shinyapps.io/irapp/, accessed 20 December 2025; version not specified) was used to perform boundary analysis on cp genomes of nine species, and the structural variation characteristics of the four boundary regions of LSC/IRB, IRB/SSC, SSC/IRA, and Ira/LSC were systematically compared.

### 2.6. Phylogenetic Analysis

To conduct a comprehensive phylogenetic analysis of *S. hookeriana* within the family Asteraceae, we downloaded the complete chloroplast genome sequences of 15 species closely related to *S. hookeriana* from GenBank. These species belong to the subfamily Cichorioideae, tribe Cichorieae, and subtribe Crepidinae: *Askellia flexuosa* (PP234598.1), *Youngia simulatrix* (PP234542.1), *Ixeridium dentatum* (OR805473.1), *Ixeris polycephala* (MK358415.1), *Crepidiastrum lanceolatum* (MK358413.1), *P. denticulata* (MK622902.1), *C. sonchifolium* (MK358414.1), *Lapsanastrum humile* (MK358416.1), *Youngia japonica* (MK358417.1), *Y. gracilipes* (MT267485.1), *S. umbrella* (MN822134.1), *Taraxacum albidum* (LC790150.2), *T. hallaisanense* (MW067130.1), *T. coreanum* (MN689809.1), and *Crepis rigescens* (OM320794.1). Additionally, we obtained the complete chloroplast genome sequences of five species from the subfamily Asteroideae to serve as outgroups: *Artemisia hedinii* (OP723217.1), *Artemisia argyi* (OP359056.1), *Ajania khartensis* (OP723181.1), *Achillea alpina* (OL684460.1), and *Abrotanella trichoachaenia* (OR069738.1). These outgroup species were chosen due to their phylogenetic distance from the ingroup taxa, ensuring sufficient sequence divergence for reliable root placement while maintaining phylogenetic relevance.

Incorporating the newly sequenced *S. hookeriana* (OM935750.1) from this study, a total of 21 Asteraceae species were used for the phylogenetic analysis. Multiple complete chloroplast sequence alignments were generated using MAFFT (version 7.526) [27] and manually refined to ensure accuracy. Phylogenetic analysis was performed using the Maximum Likelihood method implemented in IQ-TREE(version 3.0.1) [28], with the TVM+R4+FO model selected as the best-fitting model for tree construction. Support for the best tree was obtained with 1000 ultrafast bootstrap replicates (-bb 1000 -wbt -alrt 1000).

## 3. Results

### 3.1. Characteristics of S. hookeriana cpDNA

**Figure 2:**
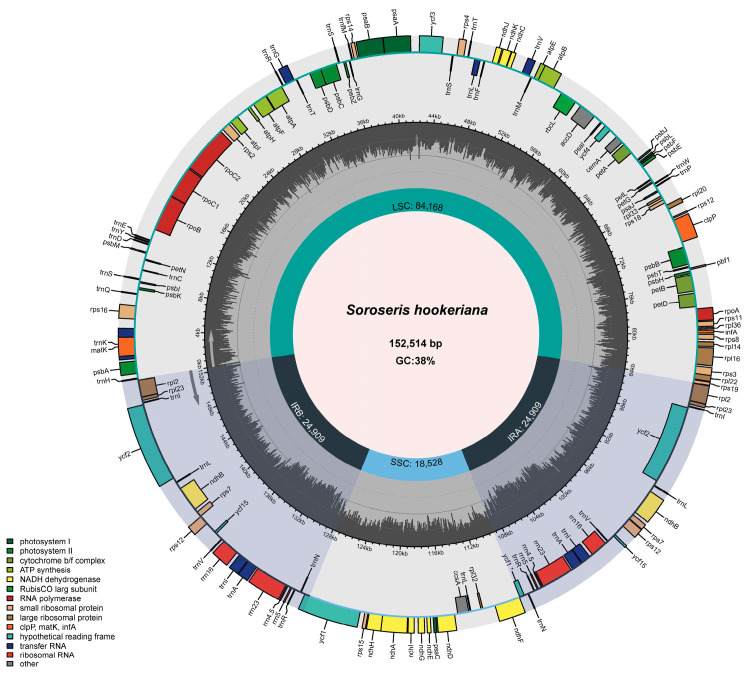
cp genome map of *S. hookeriana*.

The cp genome of *S. hookeriana* (152,514 bp) displays a typical quadripartite organization, consisting of a large single-copy region (84,168 bp), a small single-copy region (18,528 bp), and two identical inverted repeat regions (24,909 bp each) (Figure 2; Table 1). Its nucleotide composition is 31.19% T, 31.08% A, 18.78% C, and 18.94% G, yielding an overall GC content of 37.73% that varies regionally: 43.12% in IR, 35.94% in LSC, and 31.34% in SSC.

The cp genome contains 132 unique genes (excluding one pseudogene), including 87 protein-coding genes, 37 tRNA genes, and 8 rRNA genes (Table 2). Eighteen genes are duplicated in the IR regions, resulting in a total of 150 gene copies. The LSC region contains 62 protein-coding and 22 tRNA genes, the SSC region houses 12 protein-coding and a single tRNA gene, and each IR region carries 7 protein-coding, 4 rRNA, and 7 tRNA genes (in duplicate). Functionally, these genes are annotated for photosynthesis, self-replication, envelope proteins, proteases, transcription initiation factors, and other roles.

Twenty-three genes contain introns (Table 3). Among these, 21 genes possess a single intron (13 protein-coding genes and 8 tRNA genes), while two genes, *clpP* and *ycf3*, contain two introns each. The largest intron (2536 bp) is *trnK-UUU* gene whereas the smallest intron (396 bp) is *trnL-UAA* gene.

### 3.2. Codon Usage Bias

The sequences of protein-coding genes and tRNA genes were analyzed to calculate the RSCU values for each codon. A total of 62 codon types (26,165 codons) were involved in amino acid encoding (Table 4). Leucine was the most frequently encoded amino acid (2812 codons, 10.75%), whereas cysteine was encoded by the fewest codons (287 codons, 1.10%). Thirty-one codons exhibited RSCU values ≥1, of which 28 ended with A or U. Collectively, these data indicate a pronounced bias toward A/U at the third codon position in the cp genome. For methionine, RSCU values were 1.99 for AUG and 0.006 for GUG. Sequence verification confirmed that GUG occurs exclusively at gene initiation positions, functioning as a start codon for methionine. The extremely low frequency of GUG usage (representing 0.3% of methionine initiation events) demonstrates that only a minimal proportion of genes in *S. hookeriana* chloroplasts utilize GUG as the start codon. This represents a distinctive feature of the chloroplast translational system, reflecting a specialized regulatory layer and evolutionary strategy.

### 3.3. Repeat Sequence

For repeat structure analysis, 21 forward (F-type), 16 palindromic (P-type), and 2 reverse (R-type) repeats were detected in the *S. hookeriana* cp genome; no complement (C-type) repeats were found (Table 5). Repeat lengths ranged from 30 to 60 bp, with the longest repeats (60 bp) including two forward and two reverse repeats. The shortest repeats (30 bp) comprised five forward, six palindromic, and one reverse repeat. The functionally uncharacterized *ycf2* gene was predominantly associated with larger repeats (42–60 bp) in the IR regions, while photosystem I-related genes (*psaA*, *psaB*, and *ycf3*) were primarily located in the LSC region. Notably, *ycf3* exhibited broad distribution across LSC, SSC, and IR regions, which may enhance photosynthetic efficiency and contribute to the biosynthesis of bioactive compounds underlying the pharmacological properties of *S. hookeriana*.

**Figure 3:**
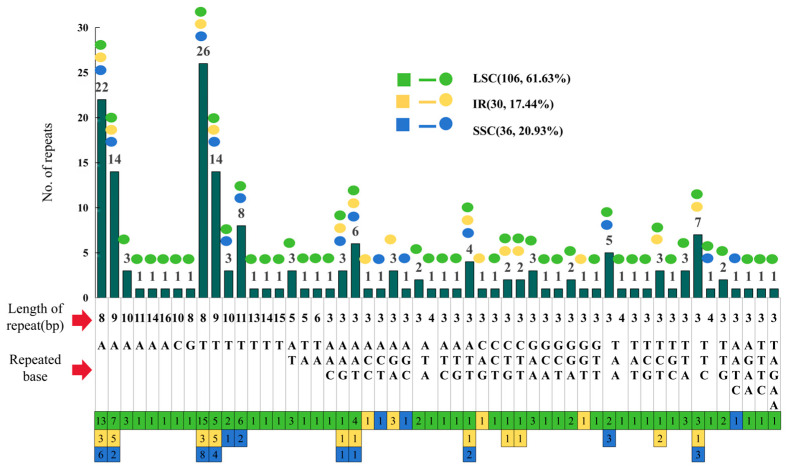
Length and distribution of SSR in the *S. hookeriana* cp genome.

The cp genome contained 172 SSR loci (Figure 3), with 106 (61.63%) distributed in the LSC region and the remainder approximately equally partitioned between IR and SSC regions (Figure 2). These SSRs comprised 98 mononucleotide repeats (56.98%), 5 dinucleotide repeats (2.91%), 65 trinucleotide repeats (37.79%), 3 tetranucleotide repeats (1.74%), and 1 pentanucleotide repeat (0.58%).

### 3.4. Comparative cp Genome Analysis and IR Boundary Analysis

**Figure 4:**
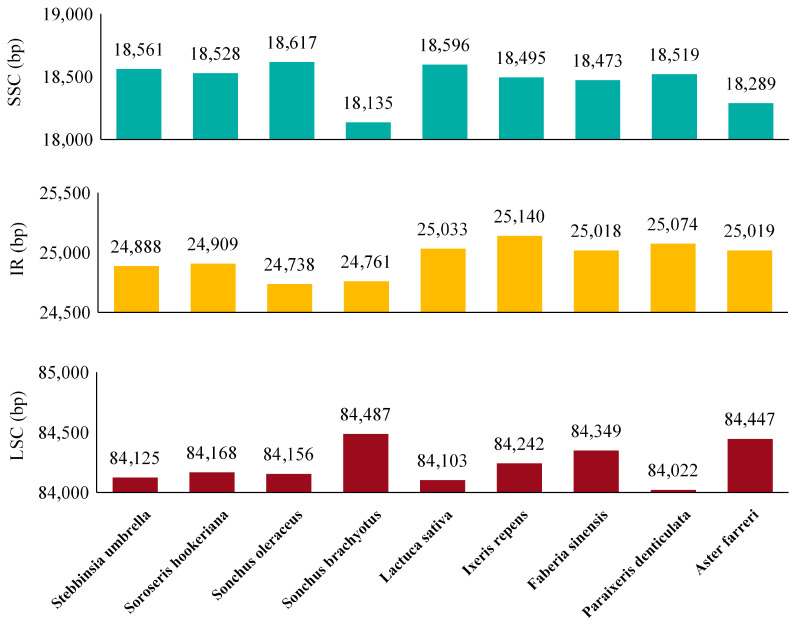
Comparison of cp genomes in Asteraceae.

cp genomes of eight Asteraceae species were retrieved from NCBI database for comparative analysis with *S. hookeriana*. Whole-genome alignment with *S. hookeriana* using mVISTA revealed that the nine cp genomes ranged from 151,849 to 153,017 bp (Figure 4) and all maintained the typical quadripartite structure. The limited size variation (1168 bp) suggests relatively constrained IR expansion/contraction during evolution in this group.

**Figure 5:**
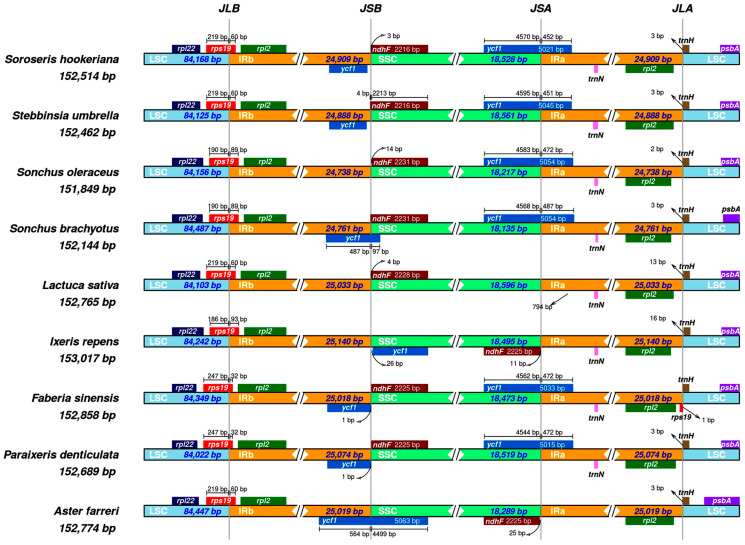
Comparison of the borders of the LSC, SSC, and IR regions among nine cp genomes.

IRScope analysis using *S. umbrella* as a reference indicated that *S. hookeriana* IR regions (24,909 bp) were 21 bp larger than those of *S. umbrella* (24,888 bp) (Figure 4 and Figure 5). The IR expansion extended 43 bp into the LSC region at the JLB junction and 33 bp into the SSC region at the JSB junction, resulting in proportional contraction of the single-copy regions. Comparative gene mapping revealed both conserved and dynamic boundary features across the nine species. The *rpl2* gene was consistently located within the IR regions (IRb and IRa) in all species, while *rpl2* remained in the LSC region, neither showing boundary displacement. In contrast, *ndhF* exhibited significant positional shifts. In *A. farreri* and *I. repens*, *ndhF* spanned the IRb/SSC boundary, shifting upstream in the JSA region at distances of 25 bp and 11 bp from the JLA junction, respectively. In *S. hookeriana*, *ndhF* completely crossed into the JSA downstream region, fully spanning the IRb/SSC region. The *ycf1* gene showed a 24 bp length reduction in *S. hookeriana* but maintained its canonical distribution across the JSA/SSC and SSC/IRa junctions. Similar patterns were observed in *S. brachyotus*, *S. oleraceus*, *P. denticulata*, and *F. sinensis*, although *ycf1* in *S. brachyotus* additionally spanned the IRb/SSC region. The *ycf1* gene was absent in *L. sativa*, shifted from upstream of JSB to downstream of JSA in *I. repens*, and was completely relocated to the IRb/SSC region in *A. farreri*.

**Figure 6:**
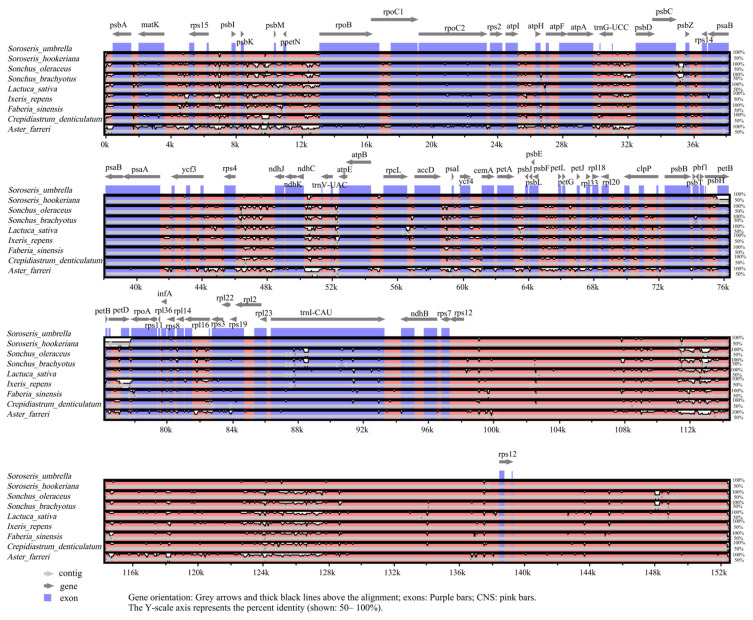
Sequence analysis of cp genome with nine Asteraceae species.

Homology visualization using mVISTA Shuffle-LAGAN mode identified the JLB and JSA boundaries as lineage-specific hotspots of variation (Figure 6). In *S. hookeriana*, the JLB boundary showed a 43 bp expansion into non-coding regions (white peaks, <70% conservation), suggesting neutral structural evolution. The JSA boundary exhibited a 21 bp expansion with low conservation (<70%) and high sequence divergence in the IRa/SSC intergenic region. Conversely, the JSB boundary displayed a 33 bp contraction with moderate conservation (70–85%) and minor rearrangement at the IRb/SSC junction. Despite these boundary shifts, essential genes including *psbA* (JLB), *ndhF* (JSB), *ycf1* (JSA), and *rpl2* (JLA) showed high conservation (red signals in mVISTA) and remained intact or functional according to IRScope analysis, indicating that boundary variations likely represent neutral structural evolution without compromising core cp genome function.

### 3.5. Phylogenetic Analyses

**Figure 7:**
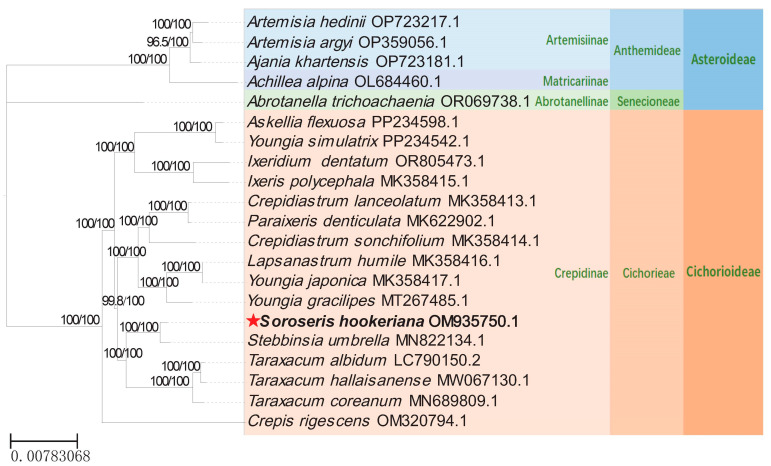
Maximum likelihood tree of 21 cp genomes of Asteraceae. Note: Phylogram obtained from the maximum-likelihood analysis of the chloroplast data on IQTree. Branch support values were obtained from 1000 ultrafast bootstrap replicates. Five species from the subfamily Asteroideae were used as outgroups, and the red pentagram indicates *S. hookeriana*, whose chloroplast genome was obtained in this study.

*S. hookeriana* and *S. umbrella* are congeneric species that formed a monophyletic clade. This clade was resolved as sister to Taraxacum species with strong bootstrap support (100%) (Figure 7). The genetic distance between *S. hookeriana* and *S. umbrella* exceeded interspecific divergence within Taraxacum, confirming that the Soroseris clade is a distinct sister lineage to Taraxacum. *S. hookeriana* and *S. umbrella* together constitute this Soroseris clade.

## 4. Discussion

*S. hookeriana* is a Tibetan medicinal plant with significant pharmacological value and a narrow endemic distribution in the Sino-Himalayan region. As an alpine specialist occupying narrow niches that may be vulnerable to climate change, it represents a candidate for future ecological monitoring studies. Understanding its genetic evolution is therefore of considerable importance [1]. This study reports the complete cp genome sequencing, assembly, and annotation of *S. hookeriana*, along with comparative and phylogenetic analyses.

The cp genome exhibits a typical quadripartite structure of 152,514 bp, consistent with most land plant cp genomes [5]. Comparative analysis with other *Soroseris* species reveals similar genome sizes and gene content, indicating relative conservation within the genus. The chloroplast genome of *S. hookeriana* contains 132 unique genes, 23 of which harbor introns. The *ycf3* gene, encoding a protein essential for photosystem I assembly [29], carries two introns of 738 bp and 699 bp. While photosynthetic activity is known to influence secondary metabolite biosynthesis in plants [30,31], the functional relevance of plastid intron size variation in *S. hookeriana* cannot be determined from genome data alone and requires future transcriptomic and functional studies.

Codon usage patterns reflect evolutionary history and can guide optimization of heterologous gene expression systems [32]. In S. hookeriana, the overall GC content is 37.73%, with region-specific values of 43.12% (IR), 35.94% (LSC), and 31.34% (SSC)—all below 45%. There are 31 codons with RSCU ≥1 in S. hookeriana cp genome, and 28 end with A/U, demonstrating a clear A/U-ending preference. These observed characteristics establish a distinct codon usage blueprint for this chloroplast genome, providing a foundation for rational heterologous gene design in this system.

RSCU analysis revealed that methionine in the cp genome of *S. hookeriana* is encoded by both AUG (RSCU = 1.99) and GUG (RSCU = 0.006). The extremely low usage frequency of GUG, accounting for only 0.3% of methionine initiation events, indicates that although the vast majority of genes initiate with AUG, a minority of genes still employ GUG as the methionine codon at the initiation site. Sequence verification confirmed that GUG occurs exclusively at the 5′ terminus of genes, representing a distinctive regulatory feature of chloroplasts compared to the nuclear genome. Previous studies have shown that in tobacco chloroplasts, GUS reporter gene expression initiated with GUG was significantly lower than that initiated with AUG [33]. This “attenuated translation” can provide low-level, precise synthesis of photosynthetic proteins (e.g., the PSII *psbC* gene product), thereby preventing resource wastage. Literature indicates that chloroplasts utilize non-AUG start codons as an additional layer of gene regulation to balance the degradation and synthesis demands of high-turnover proteins, rather than promoting overexpression [33]. Prokaryotes also employ GUG/UUG as start codons, and this conserved mechanism has persisted throughout the 1.5 billion years of chloroplast evolution, remaining highly conserved from algae to angiosperms and corroborating its cyanobacterial endosymbiotic origin. Although the complete biological significance of non-AUG start codons remains to be fully elucidated, this regulatory layer is indispensable for the dynamic balance of the photosynthetic system.

Chloroplast SSRs (cpSSRs) exhibit uniparental inheritance and are widely used for population genetics, diversity assessment, and maternity analysis [34,35]. The *S. hookeriana* cp genome contains 172 SSRs, predominantly distributed in the LSC region and comprising mainly mononucleotide and trinucleotide repeats—a pattern consistent with most plants [36]. Mononucleotide SSRs are predominantly A/T types, likely reflecting the thermodynamic stability of A-T base pairing. As cpSSRs serve as valuable markers for investigating genetic resources in medicinal plants, these SSR loci provide a molecular toolkit for population genetics and germplasm conservation of *S. hookeriana*.

Although IR regions are the most conserved portions of cp genomes, contraction and expansion at IR boundaries are common evolutionary events that contribute to size variation among cp genomes [37,38]. The nine Asteraceae species examined showed IR length variation, indicating dynamic evolutionary processes. Most species exhibited LSC expansion, except *P. denticulata* and *L. sativa*, while *S. brachyotus* and *S. oleraceus* displayed IR contraction. Length variation in boundary-associated genes such as *ycf1*, *ndhF*, and *rpl2* likely accounts for these size differences. Despite boundary shifts, the high conservation of key junction genes confirms that the *S. hookeriana* cp genome follows a relatively stable evolutionary trajectory, consistent with the theory of semi-autonomous plastid evolution.

In *S. hookeriana*, the essential gene *ycf1* spans the IR-SSC boundary, with only 452 bp of its 5021 bp length residing in IRA and 4570 bp in SSC (Figure 4). This positioning, conserved across six related Asteraceae species but absent in *L. sativa* and *A. farreri*, reveals structural plasticity of a gene whose loss is lethal in vascular plants [39]. While IR regions typically house crucial genes due to their double-copy nature, the predominant SSC localization of *ycf1* suggests a potential regulatory advantage: single-copy status may facilitate precise nuclear-plastid co-regulation [40]. This interpretation aligns with recent frameworks proposing that plastid gene repositioning shaped nuclear-plastid interaction networks during plant terrestrialization [41]. For the high-altitude specialist *S. hookeriana*, this configuration may represent a compromise between maintaining essential gene function and optimizing transcriptional responsiveness. Future nuclear transcriptome integration will directly test whether *ycf1* expression correlates with chloroplast-targeted nuclear regulators, providing insight into how genome architectural variation contributes to environmental adaptation.

Phylogenetic analysis based on cp genome sequences resolved *S. hookeriana* as sister to *S. umbrella* with robust support (bootstrap = 100), mirroring previous ITS-based phylogenies [42]. However, given the uniparental inheritance of cp genomes and limited taxon sampling, future studies should incorporate additional *Soroseris* species and nuclear markers to capture biparental evolutionary signals. Integrating complete plastid and nuclear genomic data will provide a more comprehensive understanding of the genus’s evolutionary history.

## 5. Conclusions

In this study, we characterized the complete cp genome of *S. hookeriana* (152,514 bp), which exhibits a typical quadripartite architecture harboring 132 unique genes predominantly localized in the LSC region. CUB analysis shows that natural selection is the primary force shaping its codon preferences. Comparative analysis with eight other Asteraceae species demonstrated relatively conserved plastid genome structure across the family. Phylogenetic analysis robustly supported *S. hookeriana* as sister to *S. umbrella*, with this clade forming the sister group to Taraxacum species. The comprehensive data presented in this study provide insight into the evolutionary relationships between species of the family Asteraceae, and provide an assembly of the whole cp genome of *S. hookeriana*, which may be useful for future breeding and further biological discoveries.

## Figures and Tables

**Table 1:** Basic characteristics of cp genome of *S. hookeriana*.

Nucleotide Statistics	Percentage of Bases					Length (bp)
	A (%)	C (%)	G (%)	T (%)	GC (%)	
Whole cp genome	31.08	18.78	18.94	31.19	37.73	152,514
LSC	31.86	17.68	18.26	32.20	35.94	84,168
SSC	34.67	16.34	15.00	33.99	31.34	18,528
IRA	28.62	22.33	20.80	28.25	43.12	24,909
IRB	28.25	20.80	22.33	28.62	43.12	24,909

**Table 2:** Statistical table of functional classification of chloroplast genes in *S. hookeriana*.

Category	Gene Group	Gene Name
Photosynthesis	Subunits of photosystem I	*psaA,psaB,psaC,psaI,psaJ*
	Subunits of photosystem II	*psbA,psbB,psbC,psbD,psbE,psbF,psbH,psbI,psbJ,psbK,psbL,psbM,psbT,psbZ*
	Subunits of NADH dehydrogenase	ndhA *,ndhB * (2)*,ndhC,ndhD,ndhE,ndhF, ndhG,ndhH,ndhI,ndhJ,ndhK*
	Subunits of cytochrome b/f complex	petA,petB *,petD *,petG,petL,petN
	Subunits of ATP synthase	atpA,atpB,atpE,atpF *,atpH,atpI
	Large subunit of rubisco	*rbcL*
Self-replication	Proteins of large ribosomal subunit	rpl14,rpl16 *,rpl2 * (2)*,rpl20,rpl22,rpl23*(2), *rpl32,rpl33,rpl36*
	Proteins of small ribosomal subunit	rps11,rps12 ** (2),rps14,rps15,rps16 *,rps18,rps19,rps2,rps3,rps4,rps7(2)*,rps8*
	Subunits of RNA polymerase	rpoA,rpoB,rpoC1 *,rpoC2
	Ribosomal RNAs	*rrn16*(2)*,rrn23*(2)*,rrn4.5*(2)*,rrn5*(2)
	Transfer RNAs	trnA-UGC * (2),trnC-GCA,trnD-GUC,trnE-UUC,trnF-GAA,trnG-GCC,trnG-UCC *,trnH-GUG,trnI-CAU(2)*,trnI-GAU* * (2),trnK-UUU *,trnL-CAA(2),trnL-UAA *,trnL-UAG,trnM-CAU,trnN-GUU(2)*,trnP-UGG,trnQ-UUG,trnR-ACG*(2)*,trnR-UCU,trnS-GCU,trnS-GGA,trnS-UGA,trnT-GGU,trnT-UGU,trnV-GAC*(2),trnV-UAC*,trnW-CCA,trnY-GUA,trnfM-CAU
Other genes	Maturase	*matK*
	Protease	clpP **
	Envelope membrane protein	*cemA*
	Acetyl-CoA carboxylase	*accD*
	c-type cytochrome synthesis gene	*ccsA*
	Translation initiation factor	*infA*
	other	*pbf1*
Genes of unknown function	Conserved hypothetical chloroplast ORF	*# ycf1,ycf1,ycf15*(2)*,ycf2*(2),ycf3 **,ycf4

* gene with a single intron; ** gene with two introns; # pseudogene.

**Table 3:** The length of exons and introns in genes with introns in the *S. hookeriana* cp genome.

Gene	Location	Exon I (bp)	Intron II (bp)	Exon II (bp)	Intron II (bp)	Exon III (bp)
*trnK-UUU*	LSC	37	2536	35		
*rps16*	LSC	40	856	227		
*rpoC1*	LSC	432	724	1641		
*atpF*	LSC	144	707	411		
*trnG-UCC*	LSC	23	712	47		
*ycf3*	LSC	124	738	230	699	153
*trnL-UAA*	LSC	35	396	50		
*trnV-UAC*	LSC	38	575	35		
*rps12*	IRa	114	-	232	537	26
*clpP*	LSC	71	625	292	814	228
*petB*	LSC	6	770	642		
*petD*	LSC	8	700	475		
*rpl16*	LSC	9	1053	399		
*rpl2*	IRb	390	665	435		
*ndhB*	IRb	777	669	756		
*rps12*	IRb	232	-	26	537	114
*trnI-GAU*	IRb	38	779	35		
*trnA-UGC*	IRb	38	821	35		
*ndhA*	SSC	553	1055	539		
*trnA-UGC*	IRa	38	821	35		
*trnI-GAU*	IRa	38	779	35		
*ndhB*	IRa	777	669	756		
*rpl2*	IRa	390	665	435		

**Table 4:** RSCU analysis of the amino acids in *S. hookeriana* cp genome.

Amino Acid	Codon	No.	RSCU	tRNA	Amino Acid	Codon	No.	RSCU	tRNA
Ala	GCA	419	1.1736	trnA-UGC	Asn	AAC	283	0.4412	
Ala	GCC	224	0.6276	trnA-UGC	Asn	AAU	1000	1.5588	
Ala	GCG	159	0.4452	trnA-UGC	Pro	CCA	333	1.2012	
Ala	GCU	626	1.7536		Pro	CCC	199	0.7176	
Cys	UGC	78	0.5436		Pro	CCG	161	0.5808	
Cys	UGU	209	1.4564		Pro	CCU	416	1.5004	
Asp	GAC	214	0.4068		Gln	CAA	720	1.5238	
Asp	GAU	838	1.5932		Gln	CAG	225	0.4762	
Glu	GAA	987	1.4732		Arg	AGA	490	1.8396	
Glu	GAG	353	0.5268		Arg	AGG	185	0.6948	
Phe	UUC	530	0.711		Arg	CGA	338	1.269	
Phe	UUU	961	1.289		Arg	CGC	105	0.3942	
Gly	GGA	685	1.5352	trnG-UCC	Arg	CGG	126	0.4728	
Gly	GGC	203	0.4548		Arg	CGU	354	1.329	
Gly	GGG	315	0.706		Ser	AGC	115	0.3402	
Gly	GGU	582	1.3044		Ser	AGU	408	1.2078	
His	CAC	151	0.4856		Ser	UCA	410	1.2138	
His	CAU	471	1.5144		Ser	UCC	318	0.9414	
Ile	AUA	690	0.9387		Ser	UCG	179	0.5298	
Ile	AUC	449	0.6108	trnI-GAU	Ser	UCU	597	1.767	
Ile	AUU	1066	1.4502	trnI-GAU	Thr	ACA	409	1.25	
Lys	AAA	1027	1.4682		Thr	ACC	245	0.7488	
Lys	AAG	372	0.5318		Thr	ACG	134	0.4096	
Leu	CUA	385	0.8214		Thr	ACU	521	1.592	
Leu	CUC	190	0.4056		Val	GUA	523	1.4912	trnV-UAC
Leu	CUG	185	0.3948		Val	GUC	183	0.5216	trnV-UAC
Leu	CUU	615	1.3122		Val	GUG	192	0.5472	trnV-UAC
Leu	UUA	851	1.8156	trnL-UAA	Val	GUU	505	1.4396	
Leu	UUG	586	1.2504	trnL-UAA	Trp	UGG	453	1	
Met	AUG	627	1.9936		Tyr	UAC	189	0.3826	
Met	GUG	2	0.0064		Tyr	UAU	799	1.6174	

**Table 5:** Long repeat sequences in the *S. hookeriana* cp genome.

ID	Repeat I Start	Type	Size (bp)	Repeat II Start	Mismatch (bp)	E-Value	Gene	Region
1	84,169	P	24,909	127,606	0	0	-	IR
2	91,414	F	60	91,432	0	4.92 × 10^−27^	*ycf2*;*ycf2*	IRb;IRb
3	91,414	P	60	145,192	0	4.92 × 10^−27^	*ycf2*;*ycf2*	IRb;IRa
4	91,432	P	60	145,210	0	4.92 × 10^−27^	*ycf2*;*ycf2*	IRb;IRa
5	145,192	F	60	145,210	0	4.92 × 10^−27^	*ycf2*;*ycf2*	IRa;IRa
6	56,535	F	50	56,560	−1	7.74 × 10^−19^	*rbcL*;*rbcL*	LSC;LSC
7	111,541	P	49	111,541	−1	3.03 × 10^−18^	IGS	SSC;SSC
8	54,778	F	48	54,794	0	8.26 × 10^−20^	IGS	LSC;LSC
9	74,262	P	48	74,262	−2	8.38 × 10^−16^	*pbf1*;*pbf1*	LSC;LSC
10	46,699	P	46	46,699	0	1.32 × 10^−18^	IGS	LSC;LSC
11	10,707	P	42	10,707	0	3.38 × 10^−16^	IGS	LSC;LSC
12	91,414	F	42	91,450	0	3.38 × 10^−16^	*ycf2*;*ycf2*	IRb;IRb
13	145,192	F	42	145,228	0	3.38 × 10^−16^	*ycf2*;*ycf2*	IRa;IRa
14	30,102	F	41	30,115	0	1.35 × 10^−15^	IGS	LSC;LSC
15	43,376	F	41	98,203	−2	9.98 × 10^−12^	*ycf3*;IGS	LSC;IRb
16	43,376	P	41	138,440	−2	9.98 × 10^−12^	*ycf3*;IGS	LSC;IRa
17	56,546	F	39	56,571	0	2.16 × 10^−14^	*rbcL*;IGS	LSC;LSC
18	43,378	F	39	119,515	−1	2.53 × 10^−12^	*ycf3*;*ndhA*	LSC;SSC
19	98,205	F	39	119,515	−1	2.53 × 10^−12^	IGS;*ndhA*	IRb;SSC
20	119,515	P	39	138,440	−1	2.53 × 10^−12^	*ndhA*;IGS	SSC;IRa
21	38,362	F	36	40,586	−3	2.67 × 10^−7^	*psaB*;*psaA*	LSC;LSC
22	95,161	F	35	119,518	−3	9.79 × 10^−7^	*ndhB*;*ndhA*	IRb;SSC
23	119,518	P	35	141,488	−3	9.79 × 10^−7^	*ndhA*;*ndhB*	SSC;IRa
24	30,097	F	33	30,123	−3	1.31 × 10^−5^	IGS	LSC;LSC
25	54,778	F	32	54,810	0	3.55 × 10^−10^	IGS	LSC;LSC
26	8531	F	32	35,184	−3	4.75 × 10^−5^	*trnS-GCU*;*trnS-UGA*	LSC;LSC
27	59,274	R	31	59,277	−2	5.94 × 10^−6^	IGS	LSC;LSC
28	8533	P	30	45,134	0	5.67 × 10^−9^	*trnS-GCU*;*trnS-GGA*	LSC;LSC
29	12,555	F	30	12,583	−2	2.22 × 10^−5^	IGS	LSC;LSC
30	107,071	F	30	107,103	−2	2.22 × 10^−5^	IGS	IRb;IRb
31	107,071	P	30	129,551	−2	2.22 × 10^−5^	IGS	IRb;IRa
32	107,103	P	30	129,583	−2	2.22 × 10^−5^	IGS	IRb;IRa
33	129,551	F	30	129,583	−2	2.22 × 10^−5^	IGS	IRa;IRa
34	35,186	P	30	45,134	−3	6.22 × 10^−4^	*trnS-UGA*;*trnS-GGA*	LSC;LSC
35	38,373	F	30	40,597	−3	6.22 × 10^−4^	*psaB*;*psaA*	LSC;LSC
36	67,480	F	30	99,385	−3	6.22 × 10^−4^	IGS	LSC;IRb
37	67,480	P	30	137,269	−3	6.22 × 10^−4^	IGS	LSC;IRa
38	82,407	P	30	82,409	−3	6.22 × 10^−4^	*rpl16*;*rpl16*	LSC;LSC
39	31,844	R	30	31,844	−2	2.22 × 10^−5^	IGS	LSC;LSC

## Data Availability

The cp genome sequence of *S. hookeriana* generated in this study has been deposited in NCBI GenBank under accession number OM935750.1 (Available online: https://www.ncbi.nlm.nih.gov/nuccore/OM935750.1/ (accessed on 25 December 2025)). Data will also be made available on request from the author via email (wang_jiul@163.com).
